# Potential treatment effect modifiers for manipulative therapy for children complaining of spinal pain.Secondary analyses of a randomised controlled trial

**DOI:** 10.1186/s12998-019-0282-7

**Published:** 2019-12-10

**Authors:** Kristina Boe Dissing, Werner Vach, Jan Hartvigsen, Niels Wedderkopp, Lise Hestbæk

**Affiliations:** 1 Department of Sports Science and Clinical Biomechanics, Faculty of Health Sciences, University of Southern Denmark, Campusvej 55, DK-5230 Odense M, Denmark; 2 Department of Orthopaedics and Traumatology, University Hospital Basel, Spitalstr. 21, 4031 Basel, Switzerland; 3 Nordic Institute of Chiropractic and Clinical Biomechanics, Campusvej 55, DK-5230 Odense M, Denmark; 4 Research in Childhood Health, University of Southern Denmark, SCampusvej 55, DK-5230 Odense M, Denmark; 5 Department of Orthopaedics, Sydvestjysk Sygehus Esbjerg, Finsensgade 35, DK-6700 Esbjerg, Denmark

**Keywords:** Randomised controlled trial, Effect modification, Spinal pain, Back pain, Children, Adolescents, Manipulative therapy

## Abstract

**Background:**

In children, spinal pain is transitory for most, but up to 20% experience recurrent and bothersome complaints. It is generally acknowledged that interventions may be more effective for subgroups of those affected with low back pain. In this secondary analysis of data from a randomized clinical trial, we tested whether five indicators of a potential increased need for treatment might act as effect modifiers for manipulative therapy in the treatment of spinal pain in children. We hypothesized that the most severely affected children would benefit more from manipulative therapy.

**Method:**

This study was a secondary analysis of data from a randomised controlled trial comparing advice, exercises and soft tissue treatment with and without the addition of manipulative therapy in 238 Danish school children aged 9–15 years complaining of spinal pain. A text message system (SMS) and clinical examinations were used for data collection (February 2012 to April 2014).

Five pre-specified potential effect modifiers were explored: Number of weeks with spinal pain 6 months prior to inclusion, number of weeks with co-occurring musculoskeletal pain 6 months prior to inclusion, expectations of the clinical course, pain intensity, and quality of life.

Outcomes were number of recurrences of spinal pain, number of weeks with pain, length of episodes, global perceived effect, and change in pain intensity. To explore potential effect modification, various types of regression models were used depending on the type of outcome, including interaction tests.

**Results:**

We found that children with long duration of spinal pain or co-occurring musculoskeletal pain prior to inclusion as well as low quality of life at baseline tended to benefit from manipulative therapy over non-manipulative therapy, whereas the opposite was seen for children reporting high intensity of pain. However, most results were statistically insignificant.

**Conclusions:**

This secondary analysis indicates that children more effected by certain baseline characteristics, but not pain intensity, have a greater chance to benefit from treatment that include manipulative therapy. However, these analyses were both secondary and underpowered, and therefore merely exploratory. The results underline the need for a careful choice of inclusion criteria in future investigations of manipulative therapy in children.

**Trial registration:**

NCT01504698; results

## Background

In children, spinal pain, i.e. back and/or neck pain, is transitory for most, but up to 20% experience recurrent and bothersome complaints [1, 2]. Spinal pain in adolescence is a strong predictor for similar problems in adulthood [3–5], and spinal pain ranks third among individuals living with disability within the range of 15–19 years [6]. Thus, it is important to explore the parameters that may indicate effectiveness of treatments for spinal pain in these more severely affected children. Manipulative therapy is commonly being used, despite lack of evidence of its effectiveness in children [7–9]. Current guidelines on treatment of spinal pain rely on studies of adults [10–12] and only one randomised controlled trial (RCT) including manipulative therapy for spinal pain have been conducted on children [13]. Because of the dire lack of evidence about treatment of spinal pain in children, data from existing studies should be exploited to the fullest.

For spinal pain, it is generally acknowledged that interventions may be more effective for subgroups of those affected [14, 15]. Studies of adult populations have found some variables with weak to strong evidence of a modifying effect on the treatment of spinal pain, e.g. age, expectations of treatment and quality of life [16, 17]. To our knowledge, no studies have investigated potential effect modifiers of treatment for spinal pain in children.

This study is a secondary analysis of data from an RCT investigating the effect of adding manipulative therapy to a standard treatment of advice, exercises and soft tissue treatment in Danish school children aged 9–15 years who report spinal pain [18]. In the primary analysis, we found a non-statistically significant advantage of the group not receiving manipulative therapy. Lack of significance may be due to different subgroups potentially responding differently to the interventions, it could also be due to broad inclusion criteria resulting in a heterogeneous study sample. In this paper, we therefore want to explore if we can identify potential treatment effect modifiers, i.e. certain baseline characteristics that may be associated with difference in outcomes between the two groups. Identification of such characteristics could potentially enhance clinical reasoning when selecting whether or not to include manipulative therapy in the treatment of spinal pain in children and should also be considered in future clinical trials. Because this was a small cohort, the analyses are explorative and can only be hypothesis-generating.

The aim of this study is thus to explore whether five indicators of a potential increased need for treatment act as effect modifiers for manipulative therapy in the treatment of spinal pain in children aged 9–15 years, namely:

- Number of weeks with spinal pain 6 months prior to inclusion
- Number of weeks with co-occurring musculoskeletal pain 6 months prior to inclusion
- Expectations of the clinical course
- Pain intensity at baseline
- Quality of life at baseline

## Method

### Study design

A secondary analysis of data from a pragmatic parallel observer-blinded RCT nested in a school-based open cohort.

### Setting and participants

We used data from an RCT nested in a longitudinal school-based open cohort study (CHAMPS Study-DK) [18–20]. The trial included 238 children aged 9 to 15 years reporting spinal pain from 13 Danish public schools and randomised individually from February 2012 to April 2014. The children were followed weekly with text messages (SMS) to one of their parents, inquiring about any musculoskeletal pain the child might have had during the previous week. If a parent answered positively for spinal pain, they received a standardised telephone interview regarding the complaint, which formed the basis for eligibility for the RCT and within the subsequent 2 weeks, the child was evaluated for inclusion into the trial (Table 1). Thus, there was continuous inclusion and we continued to recruit participants until 3 months prior to the end of data collection in summer 2014, resulting in varying follow-up times between 3 and 27 months.

**Table:** **Table 1**

Eligibility criteria	Inclusion criteria	Exclusion criteria
Pain was spinal and still present at the time of the interview	Spinal pain equal to or greater than 3 on an 11-box Numerical Rating Scale for more than 3 days	Serious pathology (cancer, inflammatory diseases, vertebral fractures, cauda equina syndrome)
Parent had agreed, on behalf of the child, to inclusion in the RCT		Fever and/or weight loss Nightly pain Unexplainable bruises
No manual treatment of the spine during the previous 2 months		Handicaps preventing normal physical activity

The primary aim of the RCT was to determine the effectiveness of adding manipulative therapy to other conservative care of spinal pain on both primary and secondary outcomes. Interventions included either 1) advice, exercises, and soft tissue treatment, or 2) advice, exercises, and soft tissue treatment plus manipulative therapy, and both groups were treated by licensed chiropractors. Details and results of the RCT are reported elsewhere [18, 20].

### Outcomes

Weekly positive answers on SMS to questions about spinal pain constituted the basis for the following outcomes:

1. Number of recurrences. A recurrence was defined as a positive answer of spinal pain following an answer of no spinal pain (i.e. at least one pain free week).
2. Total number of pain weeks. This was measured by the total number of weeks with positive answers of spinal pain during the follow-up period.
3. Length of episodes. This was measured by the average number of consecutive weeks with positive answers of spinal pain.

Interviews with the children 2 weeks after first treatment supplied the following two outcomes:

4. Global perceived effect (GPE). This was measured by asking the child: How will you describe your general well-being now in your neck/back as opposed to 2 weeks ago, that is before treatment started? Answers ranged from 1 to 7, with 1 being much better and 7 being much worse and were dichotomized into two groups: “Much better” (1) and “Slightly better, the same or worse” (2 to 7). By dichotomising in this way, we were quite confident that those claiming to be much better were actually much better. “Much better” was coded as 0 for the logistic regression analyses and “slightly better, the same or worse” as 1.
5. Change in pain intensity. Pain intensity was rated on an 11-point Numerical Rating Scale (NRS) with ‘0’ being ‘no pain’ and ‘10’ being ‘worst pain’ at baseline and at the interview 2 weeks later, and the difference calculated. For the regression analyses, this was changed from positive to negative (a lower number indicating a better effect) to conform with the other outcomes in the presentation of results.

### Potential treatment effect modifiers

The choice of the potential treatment effect modifiers was based on their relationship with spinal pain based on the literature as recommended by Hancock et al. [21]. In addition to variables measured at inclusion according to the protocol, we made use of existing data from the cohort and included also the number of weeks of spinal pain and co-occurring musculoskeletal pain during the 6 months prior to inclusion. We hypothesised that the most affected children would improve more with the more comprehensive treatment, i.e. including manipulative therapy, when compared to the less affected children. To make this comparison, we chose to dichotomise the variables by using the worst 10% as the cut point, thereby comparing the 10% most affected children for a given potential effect modifier to the remaining 90% for all potential modifiers.

1. *Spinal pain*. Previous studies have shown that the duration of symptoms and the number of previous episodes can predict recovery [22] and the benefit of treatment [16, 23, 24]. This variable was defined by the number of weeks with spinal pain during the 6 months prior to inclusion. Six months was chosen because we considered this to be an adequate time span for experiencing persistent or recurring pain. Spinal pain for more than 20% of the time is a considerable amount of time in pain and equalled the upper 10% of most affected children, and the variable was therefore dichotomised into ‘spinal pain less than 20% of the preceding 26 weeks’ and ‘20% or more’.
2. *Co-occurring musculoskeletal pain.* Co-occurrence of musculoskeletal symptoms predicts more persistent pain [22, 25], and therefore we considered this as a potential treatment effect modifier. Co-occurring musculoskeletal pain was defined as having pain in more than one of three regions (spine, upper and lower extremity) and as for spinal pain, pain for more than 20% of the time during the 6 months prior to inclusion was considered to be a considerable amount of pain and dichotomised accordingly. This was equal to the upper 10% of most affected children.
3. *Expectations of the clinical course*. Expectations of the clinical course has been identified as a potential effect modifier for response to treatment for low back pain in adults [16], and an association has been suggested between expectations and outcome for various treatments for musculoskeletal pain conditions [26, 27] [28],. All children were asked prior to the first treatment: “What do you expect the outcome of your spinal pain will be compared with how it is now?” This was rated on a 5-point scale with ‘1’ being ‘much worse’ and ‘5’ being ‘much better’. Children were considered as having a less favorable prognosis if they did not respond ‘better’ or ‘much better’. Thus, to assess the most affected children, the lower 10% of children was chosen as a cut point, and hence the variable was dichotomised into two groups: ‘Better’ (value = 4 and 5) and ‘Not better’ (value< 4).
4. *Pain intensity*. Pain intensity is known to predict future pain and has been shown to have moderate effect on recovery [22] and treatment effect in adults [16, 24, 29]. Pain intensity was rated on an 11-point Numerical Rating Scale with ‘0’ being ‘no pain’ and ‘10’ being ‘worst pain’. To assess the most affected children, we chose the children who had a score > 7, since we believe this equals severe pain, and hence used the upper 10% of children as the cut point. Therefore, we dichotomised them into two groups (≤7 vs. > 7), indicating low or high level of pain, respectively.
5. *Quality of life.* A low level of quality of life predicts a higher level of spinal pain [30, 31], and it has been shown to be a potential effect modifier in adults [16]. Quality of life was measured using the KIDScreen 27-item questionnaire covering five domains: Physical wellbeing, Psychological wellbeing, Autonomy and relation, Social support and peers, and School. Raw scores were transformed into T-values based on Rasch person parameter estimates with a higher score indicating higher quality of life [32]. A total sum score from the five domains was generated (KID), to have one variable displaying quality of life. This variable was also dichotomized using the 10% threshold. The decision to use a sum score is based on the assumption that the five dimensions provide similar information and are hence correlated. So, we planned to check this assumption empirically.

Finally, we wanted to explore if the number of effect modifiers in the upper 10th percentile (indicating a negative situation) displayed a dose-response relationship to treatment effect. Consequently, a variable ‘Degree of affectedness’ was generated as a sum score, by summing all potential effect modifiers into one score, ranging from 1 to 5 indicating the number of potential effect modifiers with a poor score.

### Statistical methods

The analysis included the entire cohort and followed intention-to-treat principles. A pairwise correlation was used to measure the level of association between the KID domains in order to support the choice of generating a total sum score. To ease interpretation, we used Crohnbach’s Alpha to test the reliability of this sum score, with acceptable values ranging between 0.70 to 0.95, despite the inherent uncertainties relating to this test [33, 34]. The size of a potential effect modification was explored by comparing the outcome between the two intervention groups in each of the two strata, e.g. high versus low level of pain at baseline, by using the same type of regression models as in the primary analysis (Table 2). The statistical significance of a potential effect modification was explored by conducting interaction tests for each of the potential modifiers using the same type of regression models but including the interaction between intervention group and modifier.

**Table 2:** Outcomes and statistical methods

Outcomes	Definition	Statistical method
Number of recurrences of spinal pain (3–27 months follow up) in relation to individual follow-up time	i) A positive answer on the weekly SMS for spinal pain ii) Minimum of 1 week without report of spinal pain prior to the recurrence	A hierarchical negative binomial regression model with pain free weeks included as exposure time. Intervention effects were expressed as incidence rate ratios
Length of spinal pain episodes	The number of consecutive weeks the child was affected by spinal pain	A mixed effects linear regression model with subject as random effect, outcome log transformed. Intervention effects were expressed as β-coefficient
Total number of pain weeks in relation to individual follow-up time	Total number of weeks a child was affected by spinal pain in the entire follow-up period	A hierarchical negative binomial regression model with follow-up time included as exposure time. Intervention effects were expressed as incidence rate ratio
Global perceived effect after 2 weeks	Dichotomized into two groups: “Much better” (1) and “Slightly better, the same or worse” (2 to 7).	A logistic regression model. Intervention effects were expressed as odds ratios
Change in pain intensity after 2 weeks	Rated on an 11-point Numerical Rating Scale with ‘0’ being ‘worst pain’ and ‘10’ being ‘no pain’	A linear regression model. Intervention effects were expressed as β-coefficient

An incidence rate ratio of less than 1, a β-coefficient of less than 0, or an odds ratio below 1 indicated a better outcome in the manipulative therapy group compared with the non-manipulative therapy group (low number of recurrences, short episodes and low number of total pain weeks). The interaction term isolates the impact of the modifier on the effect of the intervention treatment (manipulative therapy) versus the control treatment (non-manipulative therapy). Forest plots were made for graphical interpretation of effect estimates and the size and significance of the interaction terms. Confidence intervals and *p*-values were inspected for significance. Since the analyses are not powered for these secondary analyses, we deliberate did not focus on statistical significance, but on directions and patterns in the results and therefore tendencies of directions are reported in the summaries of the results.

To assure the relevance of generating a variable for degree of affectedness, Cohen’s kappa was used to measure the level of agreement between the potential modifiers, and again, for ease of, interpretation, we used Crohnbach’s Alpha to obtain a single estimate of the reliability of this sum score. If the measure was deemed relevant, the dose-response relationship between the degree of affectedness and the treatment effect would be estimated by using the same regression models as described above.

To investigate the overall statistical significance of our findings, we conducted a small simulation study: We generated randomly 10,000 times a subgroup covering 10% of all children and counted how often we found a treatment effect estimate favouring manipulative therapy when restricting the analysis to this subgroup. We applied this to the three outcomes „Number of recurrences “, „Length of spinal pain episode “and „Total number of pain weeks “ favouring non-manipulative therapy in the original overall analysis. We refer to the observed relative frequency as the probability to find a treatment effect favouring manipulative treatment.

STATA version 15.1 (StataCorp) was used for data analyses.

## Results

Data from 238 children were available from the original RCT and used in the analysis of the number of recurrences. Follow-up time ranged between 1 to 868 days, (mean 477 days; SD 233). For the variables spinal pain and co-occurring musculoskeletal pain prior to inclusion, 211 children fulfilled the criterion of half a year of text message answers before inclusion. There were more girls (63%) than boys, mean age at inclusion was 12.6 years, and there were 116 in the non-manipulative therapy group (49%) and 122 in the manipulative therapy group (51%).

Most of the correlations for the agreement between KID domains were in the magnitude of 0.3 to 0.6, indicating on the one side that a single domain did not drive quality of life and on the one side that all domains depict related aspects (Table 3). Crohnbachs Alpha was α = 0.81, which is considered to be good. Consequently, generating a total sum score (KID) seemed reasonable.

**Table 3:** Pairwise correlations between KID domains

	KID Phys	KID Psych	KID Auto	KID Social	KID School
KID Phys	1.0000				
KID Psych	0.5125	1.0000			
KID Auto	0.3861	0.5351	1.0000		
KID Social	0.2273	0.4856	0.919	1.0000	
KID School	0.3809	0.5834	0.5214	0.4395	1.0000

KID: quality of life questionnaire categorised into five domains (Physical wellbeing (Phys), Psychological wellbeing (Psycho), Autonomy and relation (Auto), Social support and peers (Social), and School)

**Fig. 1:**
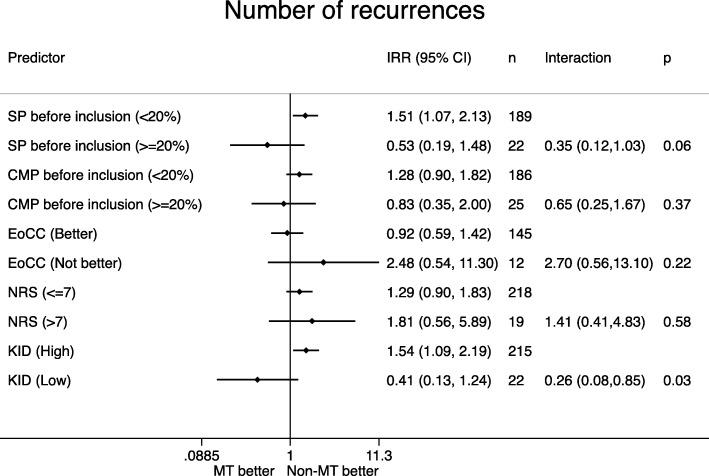
Number of recurrences. SP: spinal pain. CMP: co-occurring musculoskeletal pain. EoCC: Expectations of the clinical course. NRS: Numerical Rating Scale baseline pain intensity. KID: sum score from KIDScreen questionnaire on quality of life. IRR: incidence rate ratio. CI: confidence interval. p: *p*-value for interaction. MT: manipulative therapy. Non-MT: non-manipulative therapy

**Fig. 2:**
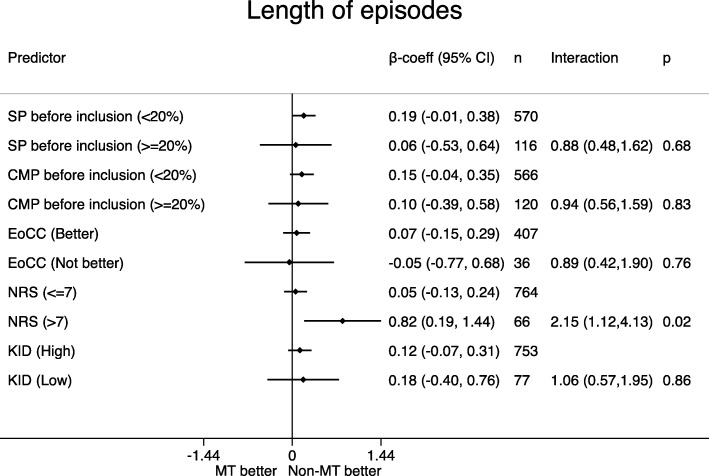
Length of spinal pain episode. SP: spinal pain. CMP: co-occurring musculoskeletal pain. EoCC: Expectations of the Clinical Course. NRS: Numerical Rating Scale baseline pain intensity. KID: sum score from KIDScreen questionnaire on quality of life. β-coeff: β-coefficient. CI: confidence interval. p: *p*-value for interaction. MT: manipulative therapy. Non-MT: non-manipulative therapy

**Fig. 3:**
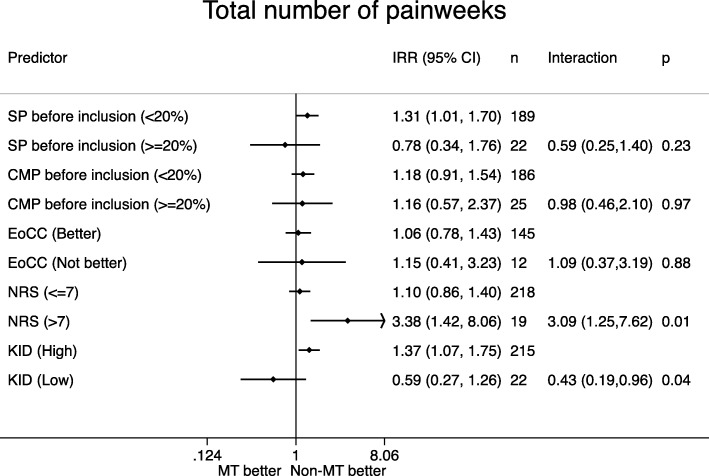
Total number of pain weeks. SP: spinal pain. CMP: co-occurring musculoskeletal pain. EoCC: Expectations of the Clinical Course. NRS: Numerical Rating Scale baseline pain intensity. KID: sum score from KIDScreen questionnaire on quality of life. IRR: incidence rate ratio. CI: confidence interval. p: *p*-value for interaction. MT: manipulative therapy. Non-MT: non-manipulative therapy

**Fig. 4:**
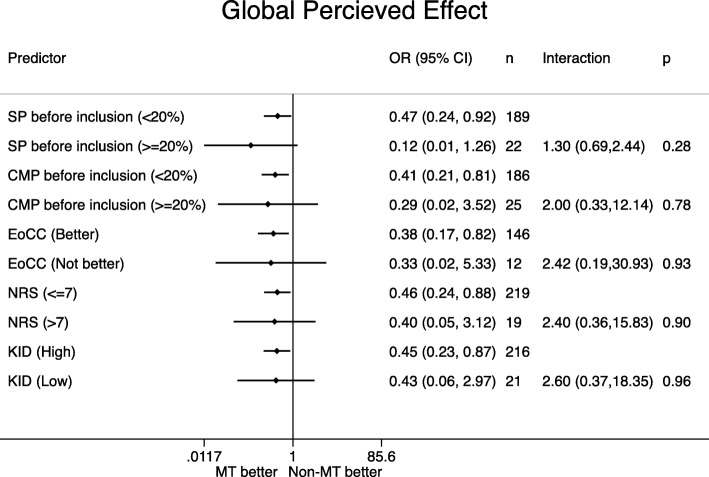
Global Percieved Effect. SP: spinal pain. CMP: co-occurring musculoskeletal pain. EoCC: Expectations of the Clinical Course. NRS: Numerical Rating Scale baseline pain intensity. KID: sum score from KIDScreen questionnaire on quality of life. IRR: incidence rate ratio. CI: confidence interval. p: p-value for interaction. MT: manipulative therapy. Non-MT: non-manipulative therapy

**Fig. 5:**
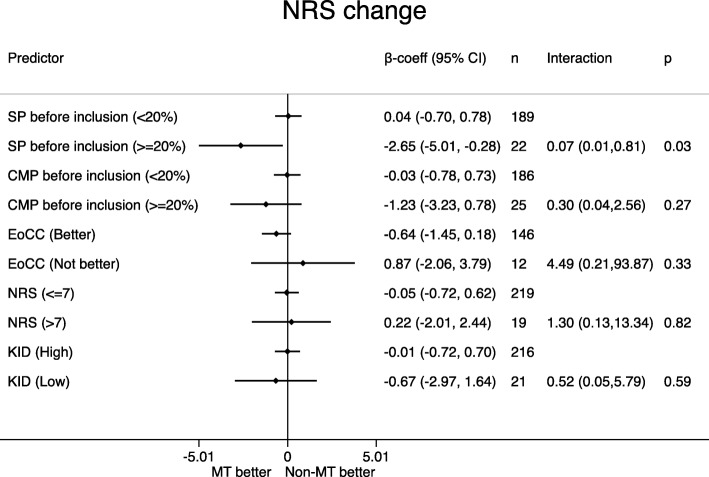
Change in pain intensity. SP: spinal pain. CMP: co-occurring musculoskeletal pain. EoCC: Expectations of the Clinical Course. NRS: Numerical Rating Scale baseline pain intensity. KID: sum score from KIDScreen questionnaire on quality of life. IRR: incidence rate ratio. CI: confidence interval. p: p-value for interaction. MT: manipulative therapy. Non-MT: non-manipulative therapy

Distribution of the potential effect modifiers in the two intervention groups can be seen in Table 4, reflecting the balance in the baseline variables. Figures 1, 2, 3, 4, 5 display all group-specific treatment effects estimates and their confidence intervals for the five outcomes, respectively. This is supplemented by estimates and *p*-values for the interactions.

**Table 4:** Distribution of baseline values of potential effect modifiers within each intervention group

	MT (*N* = 122)		Non-MT (*N* = 116)	
	*n*	%	*n*	%
**SP six months before inclusion**				
< =20% of time	103	84%	86	74%
> 20% of time	6	5%	16	14%
Missing	13	11%	14	12%
**CMP six months before inclusion**				
< =20% of time	98	80%	88	76%
> 20% of time	11	9%	14	12%
Missing	13	11%	14	12%
**EoCC**				
Better	73	60%	73	63%
Worse/same	6	5%	6	5%
Missing	43	35%	37	32%
**NRS baseline**				
< =7	111	91%	108	93%
> 7	11	9%	8	7%
Missing	0	0%	0	0%
**KID**				
High QOL	113	93%	103	89%
Low QOL	9	7%	12	10%
Missing	0	0%	1	1%

*SP* Spinal pain, *CMP* Co-occurring musculoskeletal pain, *EoCC* Expectation of the Clinical Course, *NRS* Numerical Rating Scale baseline pain intensity, *KID* Sum score from KIDScreen questionnaire on quality of life, *QoL* Quality of Life, *MT* Manipulative therapy, *Non-MT* non-manipulative therapy

### Number of recurrences (Fig. 5)

For spinal pain, co-occurring musculoskeletal pain and quality of life, the most affected children, i.e. frequent spinal pain, frequent co-occurring musculoskeletal pain and low quality of life, tended to have a better outcome in the manipulative therapy group. For pain intensity, both groups had better outcome in the non-manipulative group, but the benefit from non-manipulative therapy was largest in the most affected children. For expectations of the clinical course, the most affected children tended to have a better outcome in the non-manipulative therapy group. The *p*-values for interaction ranged between 0.03 and 0.58, and the lowest value was found for quality of life (*p* = 0.03).

### Length of spinal pain episode (Fig. 2)

A poor expectation was the only subgroup with a (slight) advantage for manipulative treatment. There were no differences between groups when looking at spinal pain, co-occurring musculoskeletal pain and quality of life. For pain intensity we observed a significant advantage for the non-manipulative therapy in children with NRS > 7. The *p*-values for interaction ranged between 0.02 and 0.86, and the lowest value was found for pain intensity (*p* = 0.02).

### Total number of pain weeks (Fig. 3)

For spinal pain and quality of life, there was a tendency for the most affected children to have a better outcome in the manipulative therapy group. For co-occurring musculoskeletal pain and expectations, we observed an advantage of non-manipulative therapy in both subgroups. For pain intensity, we again observed a significant advantage for the non-manipulative therapy in children with NRS > 7. The *p*-values for interaction ranged between 0.01 and 0.97, and the lowest value was found for pain intensity (*p* = 0.01).

### Global Percieved effect (Fig. 4)

In all subgroups we observed a better outcome in the manipulative therapy group no matter level of affectedness, but the highest benefit was seen for spinal pain. The *p*-values for interaction ranged between 0.28 and 0.96, and the lowest value was found for spinal pain (*p* = 0.28).

### Change in pain intensity (Fig. 5)

For spinal pain, co-occurring musculoskeletal pain and quality of life, there was a tendency for the most affected children to have a better outcome in the manipulative therapy group. For expectations of the clinical course we observed the opposite pattern, and there were no treatment differences in sub groups based on pain intensity. The *p*-values for interaction ranged between 0.03 and 0.82, and the lowest value was found for spinal pain (*p* = 0.03).

### Degree of affectedness

Because most of the kappa correlations between the modifiers showed poor agreement, partially indicated reverse associations (Table 5) and Crohnbachs Alpha was of low value (α = 0.35), we decided not to pursue this analysis further.

**Table 5:** Kappa coefficients between modifiers

	SP	CMP	EoCC	NRS	KID
SP	1.0000				
CMP	0.4017	1.0000			
EoCC	−0.0829	0.0042	1.0000		
NRS	0.0620	0.0448	−0.0822	1.0000	
KID	0.0005	−0.0639	0.0608	−0.0919	1.0000

*SP* Spinal pain, *CMP* Co-occurring musculoskeletal pain, *EoCC* Expectations of Clinical Course, *NRS* Numerical Rating Scale baseline pain intensity, *KID* Quality of life questionnaire

In summary, long duration of spinal pain and more co-occurring musculoskeletal complaints prior to inclusion as well as poorer quality of life may be promising variables for identifying children who may benefit from manipulative therapy in relation to several outcomes. On the other hand, a high pain intensity at baseline may be an indicator of a poorer response to manipulative therapy in this age group. Expectations to treatment showed no consistent pattern.

The probability to find a treatment effect favouring manipulative treatment in a randomly chosen subset of 10% of the children was determined for the three outcomes „Number of recurrences“, „Length of spinal pain episode “and „Total number of pain weeks“. We obtained probabilities of 28, 34, and 32%, respectively. Hence, we must be aware that at least some of our findings may reflect chance results.

## Discussion

To our knowledge, this is the first study that has tried to identify potential treatment effect modifiers when comparing two different conservative interventions for spinal pain in school children.

Our overall hypothesis was that children being worse off at baseline would have a better outcome if randomised to the manipulative therapy group. This could not be confirmed in these secondary analyses, but they did support the hypothesis for spinal pain prior to inclusion, quality of life, and for co-occurring musculoskeletal pain prior to inclusion.

We found some indications that low quality of life can predict a benefit from manipulative therapy. We know from other studies that quality of life is associated with spinal pain in children, and Dolphens et al. [31] found that the comorbid pain domain followed by the physical domain were particularly important. Balague et al. [35] reported that low back pain marginally affects quality of life, but a subgroup of children with both low back pain and whole-body pain had significantly impaired quality of life. Since quality of life furthermore has been found to affect treatment in adults, we believe, that quality of life should be considered for further exploration as a potential effect modifier in future trials dealing with treatment of spinal pain in children.

Other studies have demonstrated the importance of taking expectations into account when looking at treatment effect, and higher expectations usually predict a better outcome [27, 28, 36]. We did not see any indications of this in relation to manipulative therapy, and thus this may measure a different construct, possibly only weakly related to the degree of physical symptoms. The prognostic ability of expectations in adults might also rely on experiences which the children have not yet accumulated, However, it might also be an inherent weakness in our data, since most children had positive expectations, and we therefore had to include ‘the same’ in the ‘poor expectations’ category to reach the 10%.

A low level of spinal pain has been associated with a better outcome [24], but high levels of pain provide greater potential for improvement. Therefore, we had expected to find a modifying effect in the same direction as the other variables but found the opposite with statistical significance for length of episodes and total number of pain weeks. Hence children with a high level of pain may simply not benefit from manipulative treatment. In the main study, there was no significant difference in change in pain intensity between the two intervention groups even though children in the manipulative therapy group indicated statistically significant better global perceived effect. However, some children reported to feel better, although reporting a higher score on the Numerical Rating Scale at follow up than at baseline. This indicated that pain assessment has to be interpreted with care in this population and may also explain the unexpected result. Note that several studies have validated the Numerical Rating Scale in paediatric samples in general [37, 38] .

### Strengths and limitations

The primary strength of this study is that it is based on data from an RCT, which is considered to be the definitive type of data to explore effect modification, and we limited a priori the number of potential effect modifiers included to minimise the risk of spurious findings [21]. Secondly, we have weekly data, which gives a very complete picture of the outcomes. Some of the potential effect modifiers were affected by missing values (Table 3), but they were equally distributed between intervention groups and hence are unlikely to bias the investigation of effect modification.

Our trial was clearly underpowered for this type of analysis and therefore our results can at best be regarded as hypothesis-generating [39]. However according to Pincus [40], most datasets are under-analysed in back pain trials and therefore post-hoc analysis should be cautiously supported. We therefore followed the methodological criteria for exploring modification effect by Pincus [40]: modifiers should be measured prior to randomisation, measurement of baseline factors should be of adequate quality, and the analysis should include an explicit interaction test.

We are aware that multiple testing can lead to spurious findings. Furthermore, we recognise that by dichotomising variables, we also reduce the potential information available from these variables. We have noticed a great variety in how studies on effect modification have been analysed [41–43], and we chose this approach to be able to compare the most affected with the less affected children and to facilitate interpretation of the results.

Our study is exploratory and the results from the simulation study underlines that our findings may at least partially have occurred by chance, and hence require validation in independent studies.

## Conclusion

We found that children with long duration of spinal pain and co-occurring musculoskeletal pain prior to inclusion and low quality of life tended to benefit from management that included manipulative therapy over management not including manipulative therapy, whereas the opposite was seen for children reporting high intensity of pain. Clinically, this could indicate that the more comprehensive treatment regime should primarily be offered to children most affected by these baseline characteristics. In future RCTs aiming to study effectiveness of manipulative therapy in children, this information may inform inclusion criteria.

## Data Availability

Data are from the Childhood Health, Activity and Motor Performance School Study (CHAMPS Study-DK) and are available on request from the project manager Niels Wedderkopp.
